# Supplementary figures and images for: The accessory protein CvnF8 modulates histidine kinase activity in an actinobacterial G protein system in Streptomyces coelicolor
Source: mBio. 2026 May 27;17(7):e00774-26. doi: 10.1128/mbio.00774-26 (PMC13343842; doi:10.1128/mbio.00774-26)

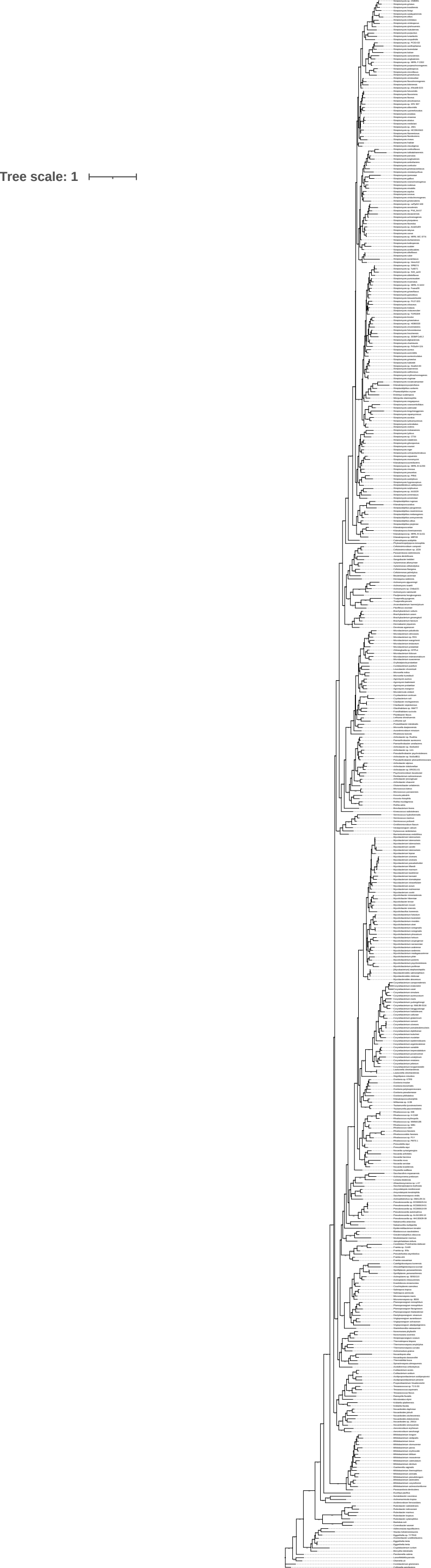

Supplement: Data File S2 — ribo_partition_species_tree_bootstraps. [file mbio.00774-26-s0002.pdf]

Free scale: 10

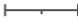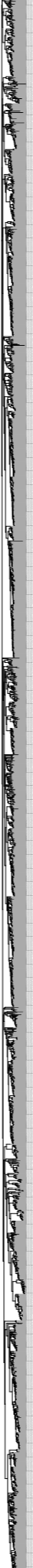

Supplement: Data File S7 — CvnA tree with genome/gene numbers. [file mbio.00774-26-s0007.pdf]
