# Supplementary material for: The accessory protein CvnF8 modulates histidine kinase activity in an actinobacterial G protein system in Streptomyces coelicolor
Source: mBio. 2026 May 27;17(7):e00774-26. doi: 10.1128/mbio.00774-26 (PMC13343842; doi:10.1128/mbio.00774-26)
Supplement: Supplemental Figures and Tables — Fig. S1-S14; Tables S1 and S2. [file mbio.00774-26-s0009.pdf]

Supplementary Information for:

The accessory protein CvnF8 modulates histidine kinase activity in an Actinobacterial G protein system in *Streptomyces coelicolor*

Luis M. Cantu Morin, Kilian Dekoninck, Kyung-Yoon Min, and Matthew F. Traxler*

Department of Plant and Microbial Biology, University of California, Berkeley, California, USA

Contents

Supplementary Figures

Supplementary Figure 1. **Species phylogeny and distribution of Actinobacterial G protein systems (AGPSs) across 485 Actinomycetota genomes.**

Supplementary Figure 2. **Correlation between genome size and Actinobacterial G protein systems (AGPSs) count across 485 Actinomycetota genomes.**

Supplementary Figure 3. **Structural prediction confidence for Alphafold model of a dimer of CvnA8 with two monomers of CvnF8.**

Supplementary Figure 4. **Comparison of CvnA8/F8 with CvnA7/F7 and CvnA11/E11 AlphaFold2 predictions.**

Supplementary Figure 5. **Non-cognate CvnAX/CvnF8 AlphaFold2 predictions.**

Supplementary Figure 6. **Replicate experiments demonstrating co-purification of His_6–$CvnA8_{81–505}$ and Strep–CvnF8.**

Supplementary Figure 7. **Purification of Strep-CvnA8.**

Supplementary Figure 8. **Concentration of Strep-CvnA8 via Gel Filtration.**

Supplementary Figure 9. **Purification of Strep-CvnA8(H183A) and Strep-CvnA8(H163A)**

Supplementary Figure 10. **Purification of Strep-CvnF**

Supplementary Figure 11. **ATPase activity of CvnA8 and CvnA8+CvnF8**

Supplementary Figure 12. **Epistatic effects on lanthipeptide biosynthetic gene expression by the Actinobacterial G protein systems (AGPSs) components CvnA8 and CvnF8.**

Supplementary Figure 13. **Epistatic effects on lanthipeptide biosynthetic gene expression by the Actinobacterial G protein systems (AGPSs) components CvnA8 and CvnB8.**

Supplementary Figure 14. **Epistatic effects on lanthipeptide biosynthetic gene expression by the Actinobacterial G protein systems (AGPSs) components CvnF8 and CvnB8.**

Supplementary Tables

Supplementary Table 1. Strains used in this study.

Supplementary Table 2. Primers used in this study.

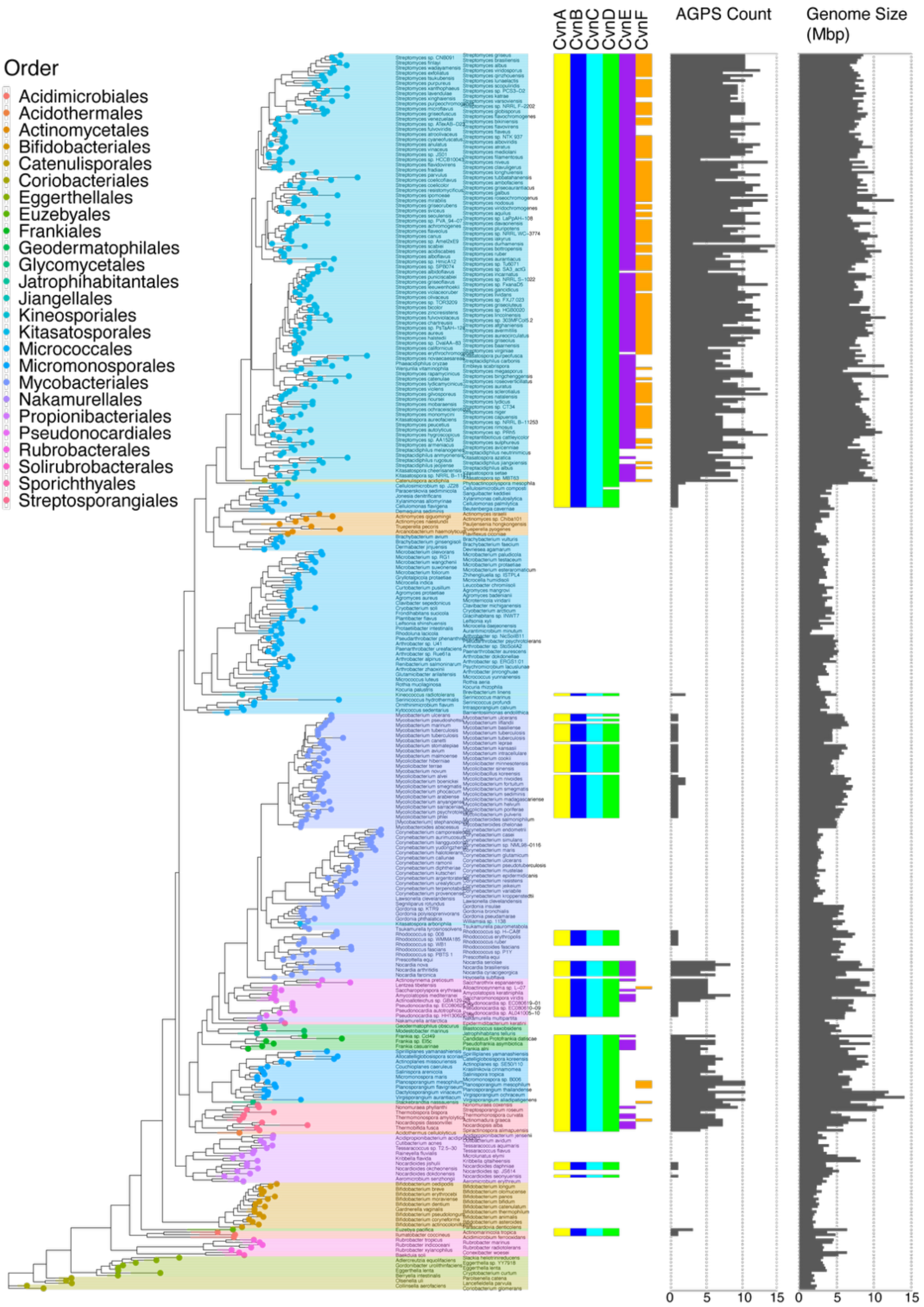

Fig. S1. Species phylogeny and distribution of Actinobacterial G protein systems (AGPSs) across 485 Actinomycetota genomes. A maximum-likelihood species tree was constructed from

485 representative genomes within the phylum Actinomycetota. A total of 289 genomes encode at least one copy of an AGPS. The presence of syntenic orthologs from each AGPS gene cluster (CvnA, CvnB, CvnC, and CvnD) is shown as a binary heatmap. Two additional tracks summarize genome features: the AGPS Count bar plot indicates the total number of AGPSs per genome, and the Genome Size bar plot reports the total genome size (in megabase pairs, Mbp). Tree tips are colored by taxonomic order to highlight lineage-specific trends in AGPS distribution and genomic features.

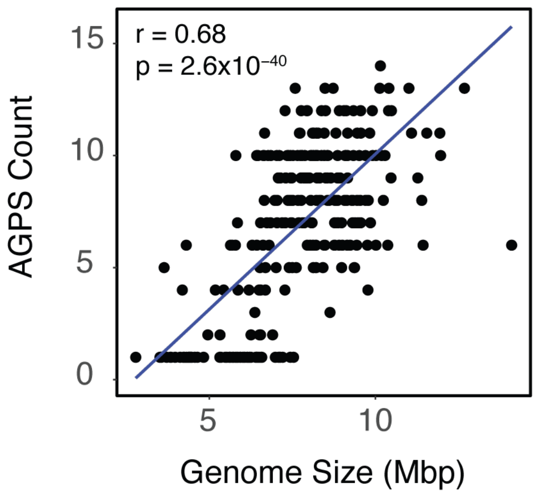

Fig. S2. Correlation between genome size and Actinobacterial G protein systems (AGPS) count across 485 Actinomycetota genomes. Each point represents a genome in the Actinomycetota species tree (see Fig. S1). The number of AGPSs detected in each genome is plotted against total genome size (in megabase pairs, Mbp). A significant positive correlation was observed between genome size and AGPS count (Pearson's $r = 0.68$, $p = 2.6 \times 10^{-40}$), suggesting that genomes with larger coding capacity tend to harbor more AGPS loci.

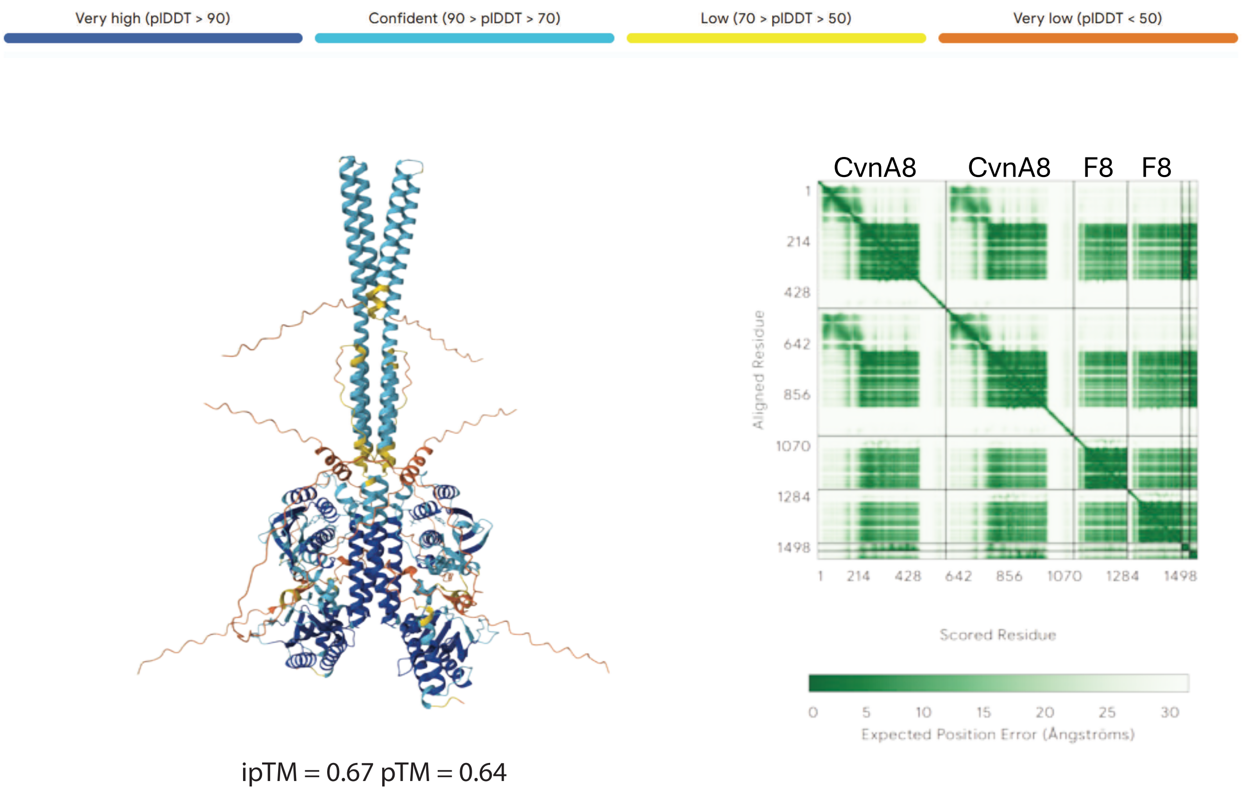

Fig. S3. Structural prediction confidence for Alphafold model of a dimer of CvnA8 with two monomers of CvnF8. Colors correspond to the confidence intervals shown at top. Note that CvnA8 contains a long unstructured region at the C-terminus which is not shown in the structural model, but is included in the heat map at left.

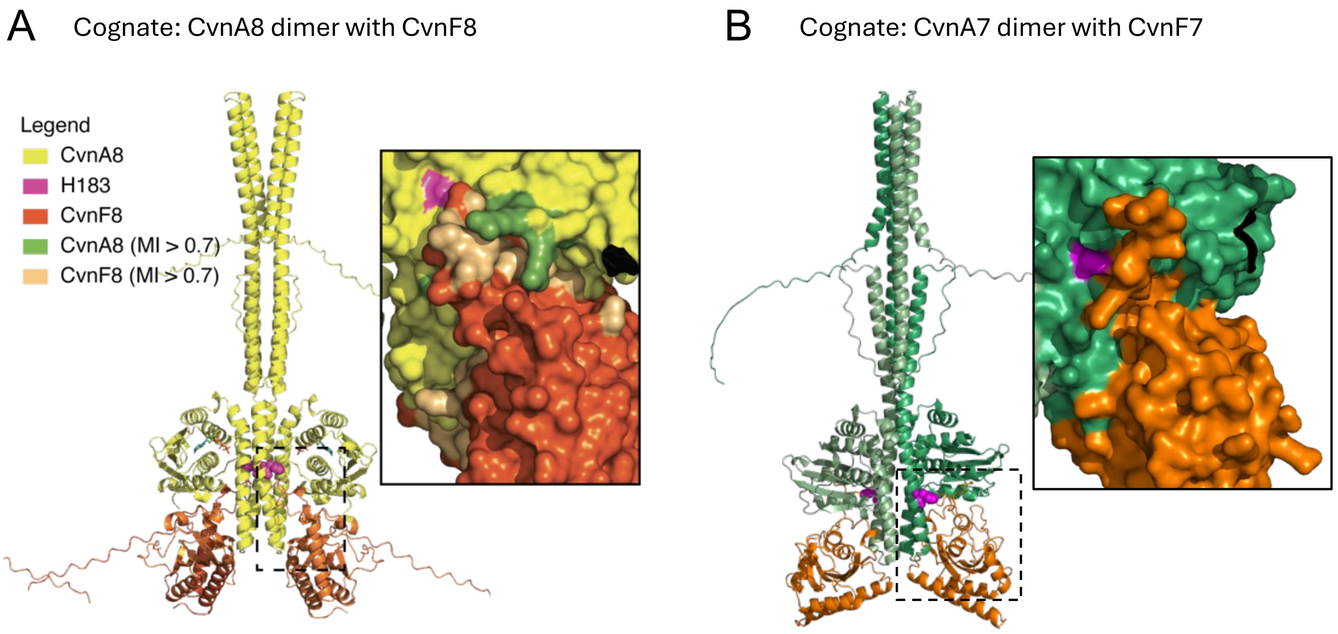

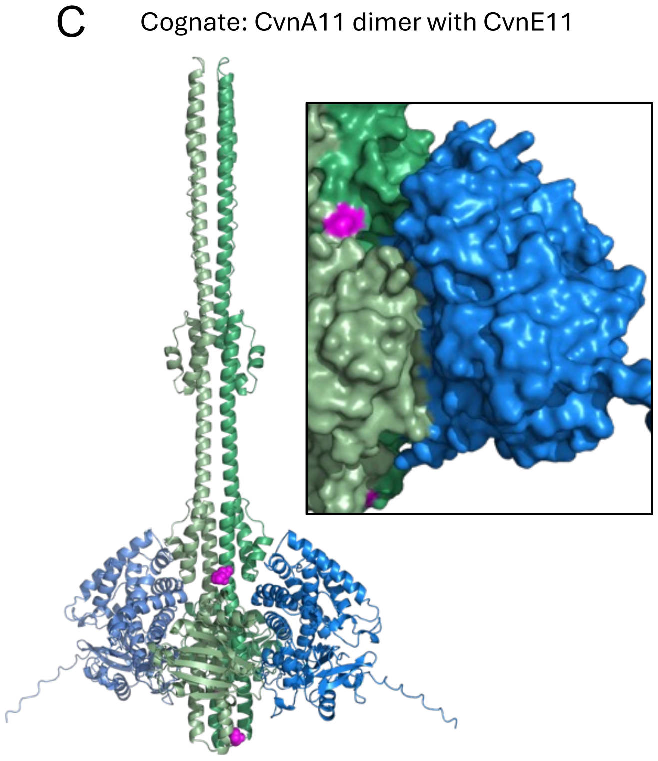

Fig. S4. Comparison of CvnA8/F8 with CvnA7/F7 and CvnA11/E11 AlphaFold2 predictions. (A) Dimer of CvnA (yellow) with two CvnF8 monomers engaged below the ATPase domains, as shown in Fig. 4 of the main text (AlphaFold 3 prediction). (B) Dimer of CvnA7 (green) with two CvnF7 monomers. A histidine at a possible site of autophosphorylation is highlighted in magenta in both CvnA7 monomers. AlphaFold2 places CvnF7 in a similar position compared to CvnF8 in (A). (C) Dimer of Cvn11A with two monomers of CvnE11 (putative cytochrome P450). Two histidine residues in the DHp domain of the dark green Cvn11A monomer are highlighted in magenta. Long unstructured regions at the C-terminal ends of CvnA proteins are hidden for clarity.

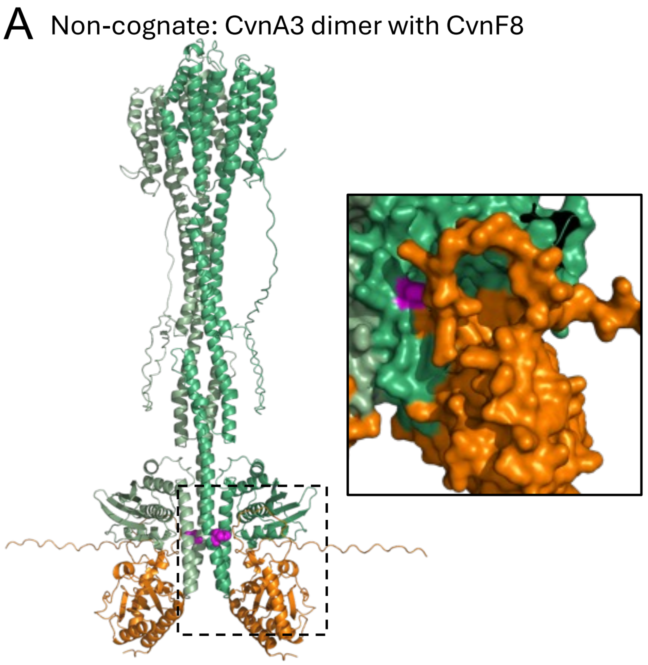

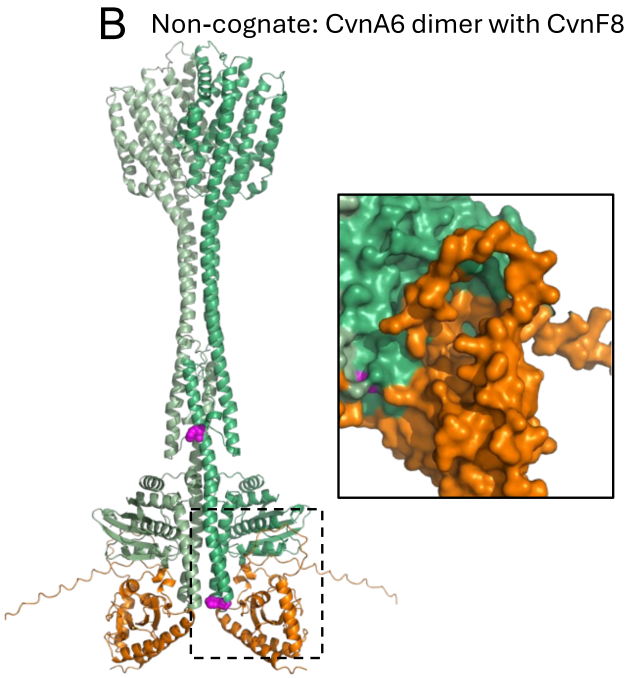

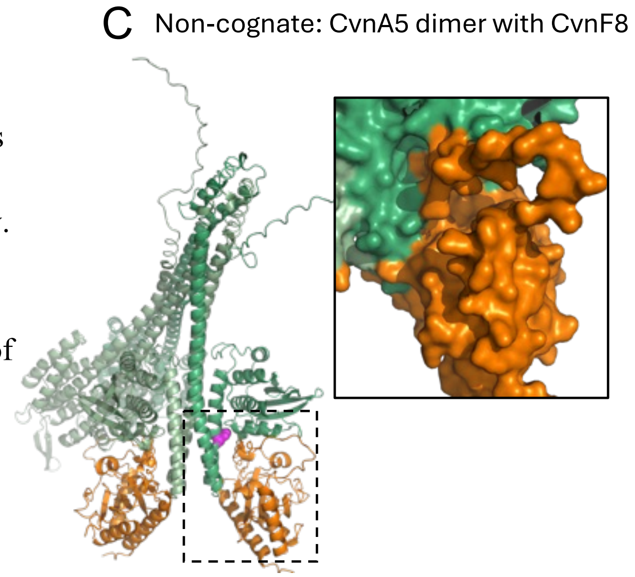

Fig. S5. Non-cognate CvnAX/CvnF8 AlphaFold2 predictions. AlphaFold2 places CvnF8 (orange) in a similar position and orientation with non-cognate CvnAs (green) compared to when it is included with its cognate CvnA8 (Fig S4A), but elements of the CvnF8 unstructured N-terminal region are arranged differently. (A) Dimer of CvnA3 with two CvnF8 monomers. A histidine at a possible site of autophosphorylation is highlighted in magenta in both monomers. (B) Dimer of CvnA6 with two CvnF8 monomers. Two histidine residues in the DHp domain of the dark green Cvn6A monomer are highlighted in magenta. (C) Dimer of CvnA5 (green) with two CvnF8 monomers. The sensory/transmembrane domains of the CvnA5 dimer are folded down behind the other domains. It is anticipated that membrane insertion of CvnA5 would lead to a normal straight configuration as shown for the other histidine kinases throughout. A histidine in the DHp domain of the dark green CvnA5 monomer is highlighted in magenta. Long unstructured regions at the C-terminal ends of CvnA proteins are hidden for clarity.

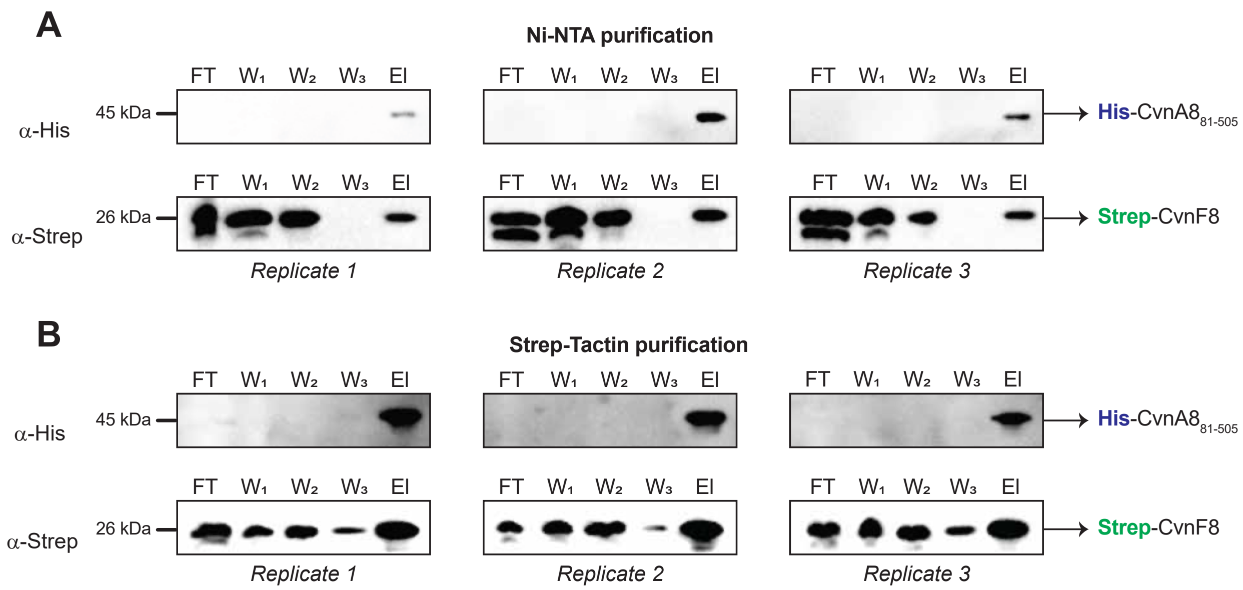

Fig. S6. **Replicate experiments demonstrating co-purification of His_6–CvnA8$_{81–505}$ and Strep–CvnF8.** The truncated His_6-tagged CvnA8 (residues 81–505, ~45 kDa) and full-length Strep-tagged CvnF8 (~26 kDa) were co-expressed from a single plasmid (pKiD45) in *E. coli* BL21(DE3). Pull-downs were performed from cell lysates using either Ni-NTA resin (Thermo Fisher Scientific) for the His_6-tag (Panel A) or Strep-Tactin Sepharose (IBA Lifesciences) for the Strep-tag (Panel B). Flow-through (FT), washes (W1–W3), and elution (El) fractions were analyzed by Western blot with monoclonal anti-His or anti-Strep antibodies.

(A) Ni-NTA pull-downs (Replicates 1–3): His_6–CvnA8 was used as bait. In all replicates, His_6–CvnA8 is detected exclusively in the elution, while Strep–CvnF8 is lost in FT and early washes but consistently co-elutes with His_6–CvnA8, indicating stable interaction. Replicate 2 is identical to the one shown in the main figure (Figure X).

(B) Strep-Tactin pull-downs (Replicates 1–3): Strep–CvnF8 was used as bait. Strep–CvnF8 is present in all fractions due to resin saturation, with decreasing signal in later washes and recovery in the elution. His_6–CvnA8 is only observed in the elution fractions, confirming its co-elution across replicates. Replicate 2 is the same as shown in the main figure (Fig. 5).

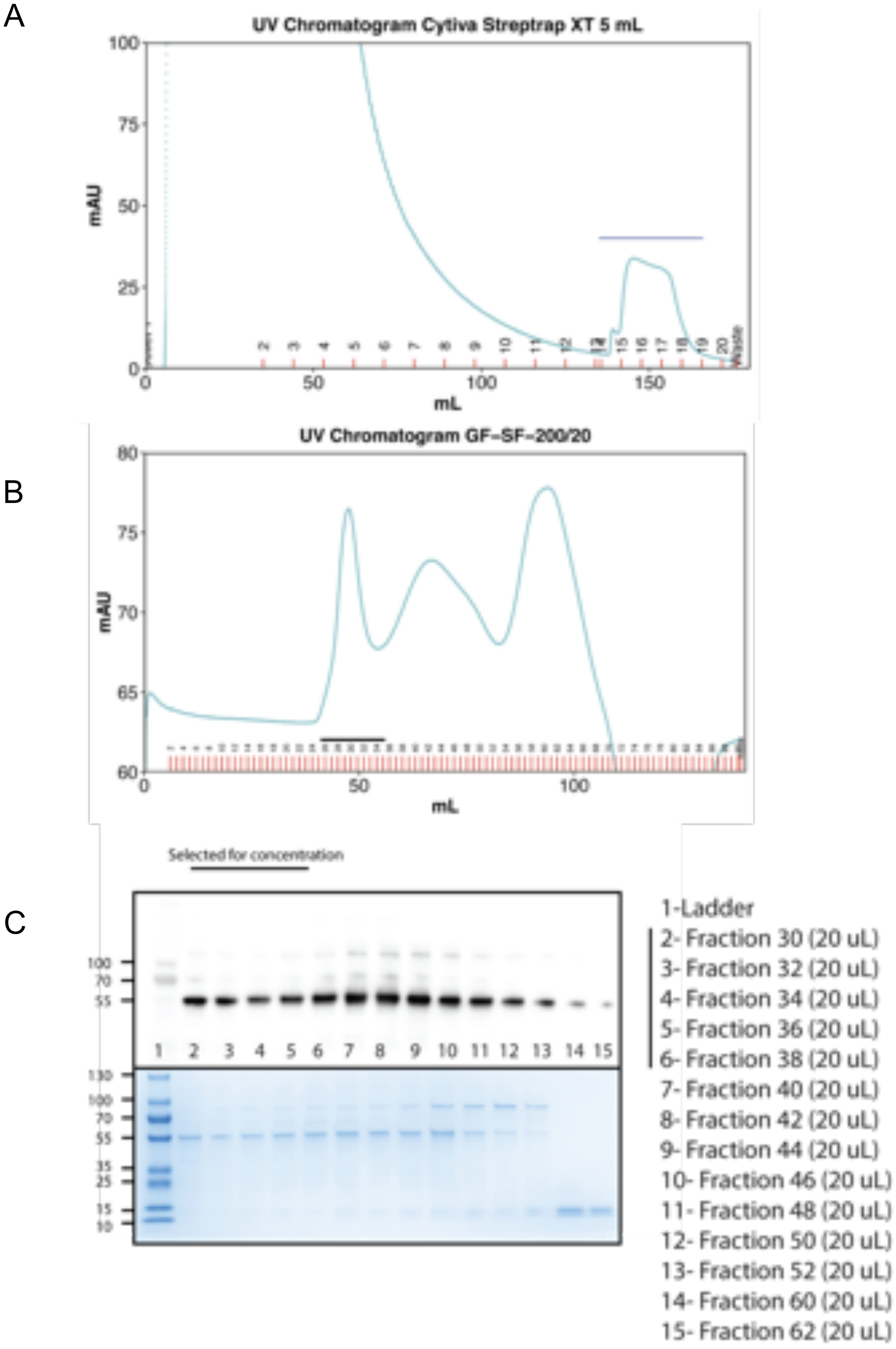

Fig. S7. Purification of Strep-CvnA8. (A) Affinity chromatography of Strep-tagged CvnA8 using a 5 mL Strep-Tactin XT column (Cytiva, #29401322). (B) Size-exclusion chromatography profile of the affinity-purified protein. (C) SDS-PAGE gel showing elution fractions from gel filtration; fractions 30–38 were pooled and concentrated for downstream applications.

A

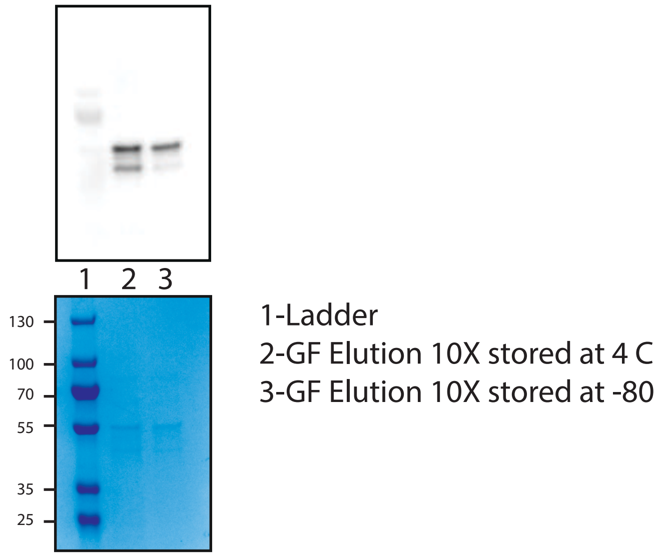

Fig. S8. Concentration of Strep-CvnA8 via Gel Filtration. (A) Concentration of Strep-tagged CvnA8 using a Gel filtration column (Superdex 75 Increase GL 10/300, from Cytiva).

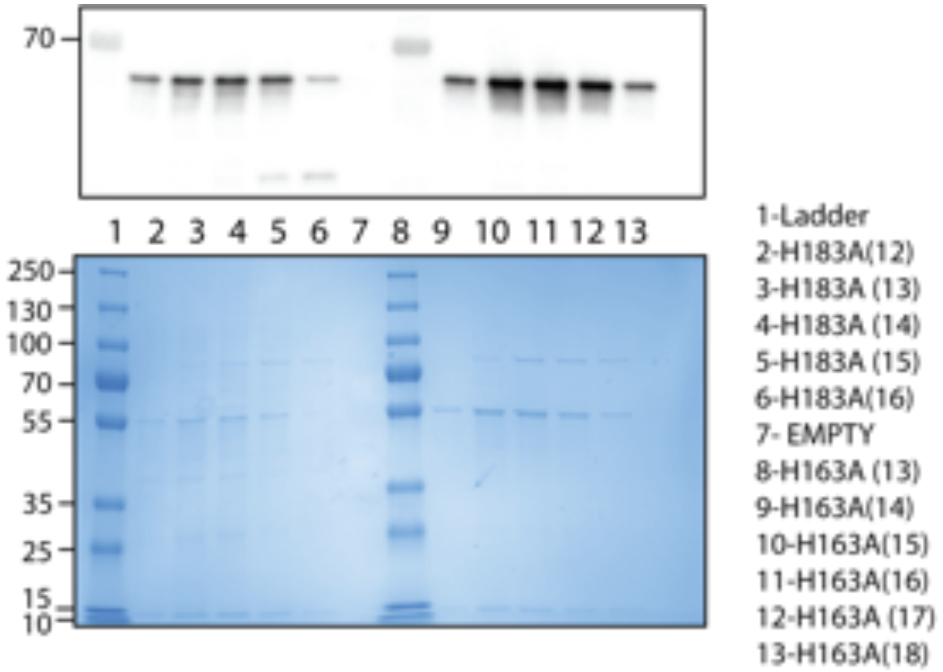

Fig. S9. Purification of Strep-CvnA8(H183A) and Strep-CvnA8(H163A). SDS-PAGE gel showing elution fractions from gel filtration; fractions in wells 2 and 9 were collected and concentrated for downstream applications.

A

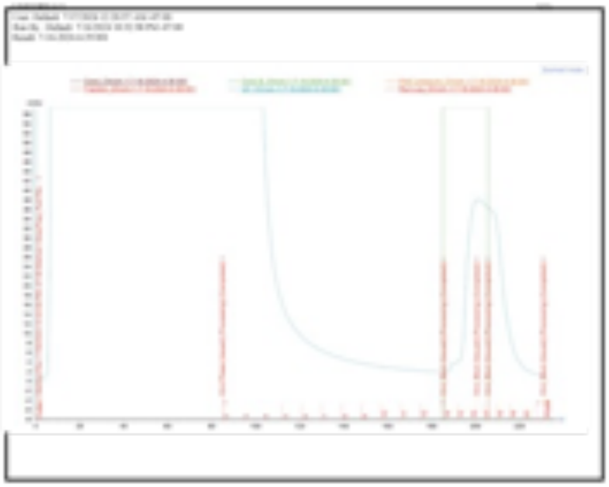

B

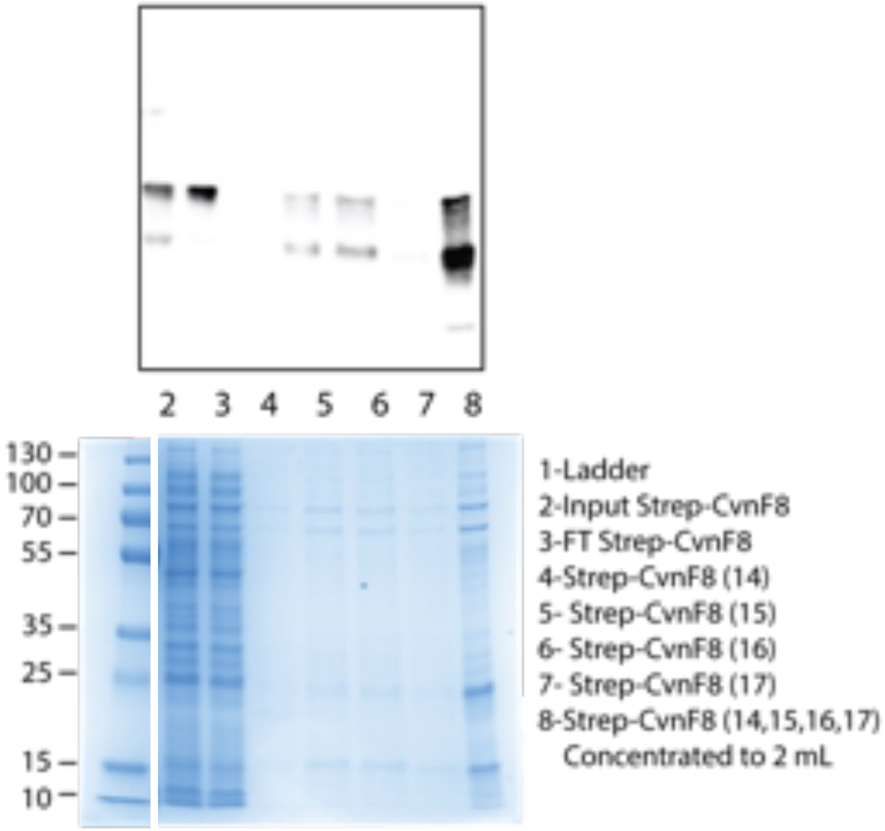

Fig. S10. Purification of Strep-CvnF. (A) Affinity chromatography of Strep-tagged CvnF8 using a 5 mL Strep-Tactin XT column (Cytiva, #29401322). (B) SDS-PAGE gel showing elution fractions from gel filtration; fractions in wells 8 were selected for downstream applications.

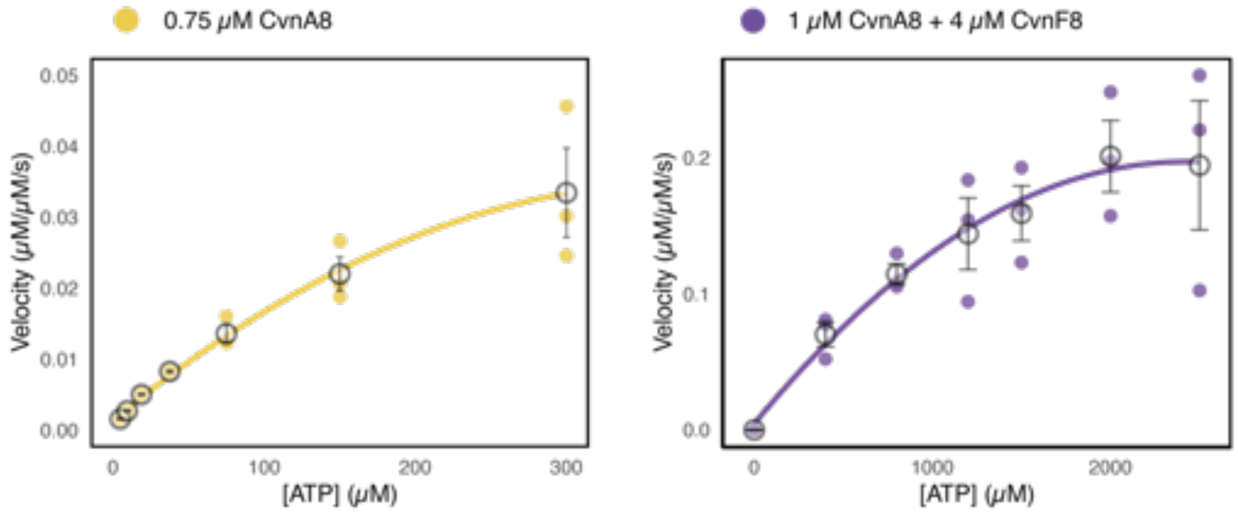

Fig. S11. ATPase activity of CvnA8 and CvnA8+CvnF8. ATP hydrolysis was measured using a luciferase-based luminescence assay (Kinase-Glo) at varying ATP concentrations for CvnA8 (0.75 µM, yellow) and the CvnA8-CvnF8 complex (1 µM CvnA8 + 4 µM CvnF8, purple). Reactions were incubated for 60 minutes before signal detection. Velocity (µM/µM/s) was calculated by normalizing ATP consumption to enzyme concentration and incubation time. Each data point represents the mean of technical replicates, and error bars indicate standard error. Michaelis-Menten fits were applied to calculate kinetic parameters, which are reported in Table 1.

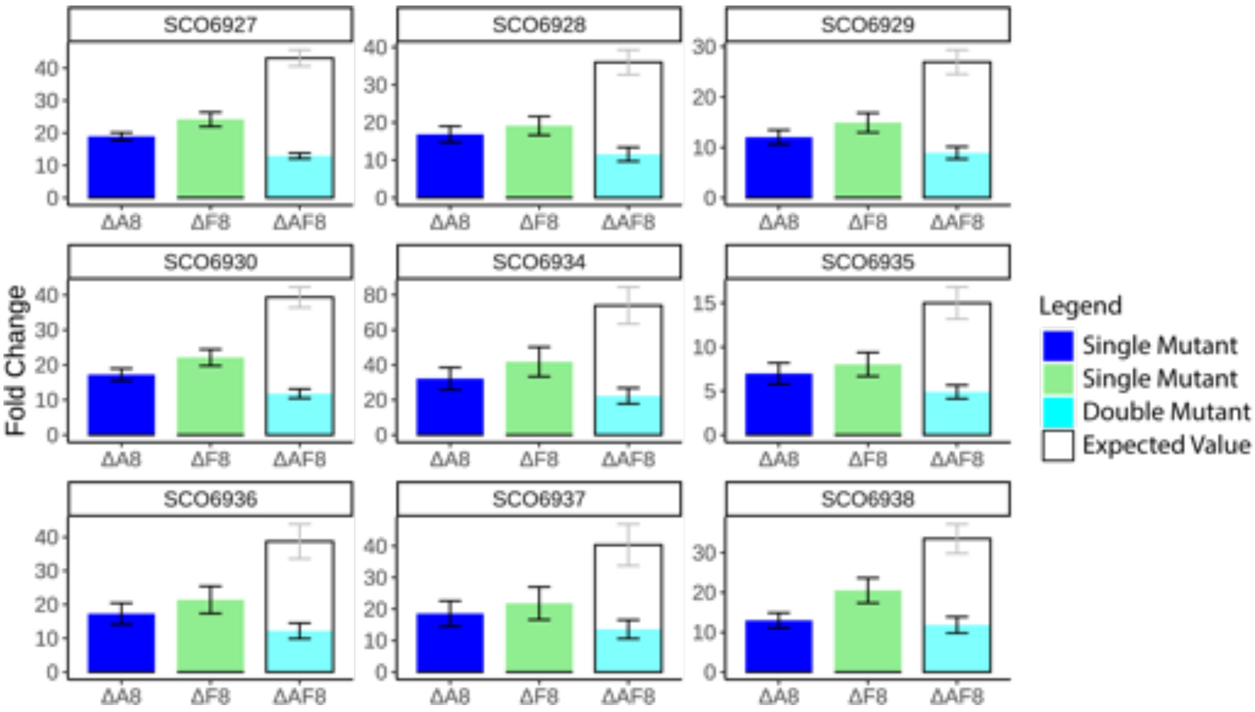

Fig. S12. Epistatic effects on lanthipeptide biosynthetic gene expression by the Actinobacterial G protein systems (AGPSs) components CvnA8 and CvnF8. Fold-change expression data from Nanostring profiling of nine genes within a lanthipeptide biosynthetic gene cluster associated with the Cvn8 AGPS. Bar plots show expression levels in single mutants (*ΔcvnA8*, *ΔcvnF8*) and the *ΔcvnA8ΔcvnF8* double mutant. Hollow bars indicate the expected additive fold-change in the double mutant based on individual mutant effects. In all cases, observed expression in the double mutant deviates from the additive expectation, indicating epistatic (non-additive) effects of CvnA8 and CvnF8 on transcriptional output of the cluster. Note: *ΔcvnA8* and *ΔcvnF8* refer to full-gene deletions generated by replacement of the open reading frame with an apramycin resistance cassette.

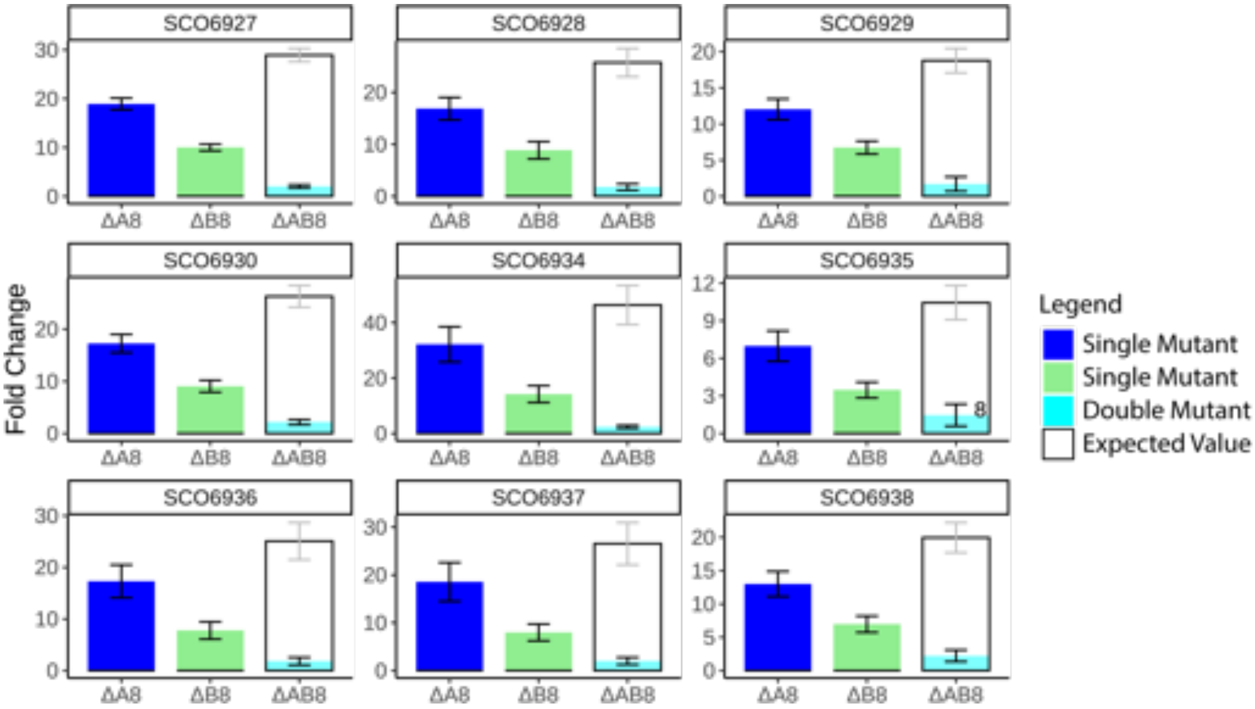

Fig. S13. Epistatic effects on lanthipeptide biosynthetic gene expression by the Actinobacterial G protein systems (AGPSs) components CvnA8 and CvnB8. Fold-change expression data from Nanostring profiling of nine genes within a lanthipeptide biosynthetic gene cluster associated with the CvnA8/CvnB8 AGPS. Bar plots show expression levels in single mutants (*ΔcvnA8*, *ΔcvnB8*) and the *ΔcvnA8ΔcvnB8* double mutant. Hollow bars represent the expected additive fold-change in the double mutant based on single mutant values. Deviations between observed and expected expression in the double mutant reveal epistatic (non-additive) effects of CvnA8 and CvnB8 on regulation of the cluster.

Note: ΔcvnA8 and ΔcvnB8 refer to full-gene deletions generated by replacement of the open reading frame with an apramycin resistance cassette.

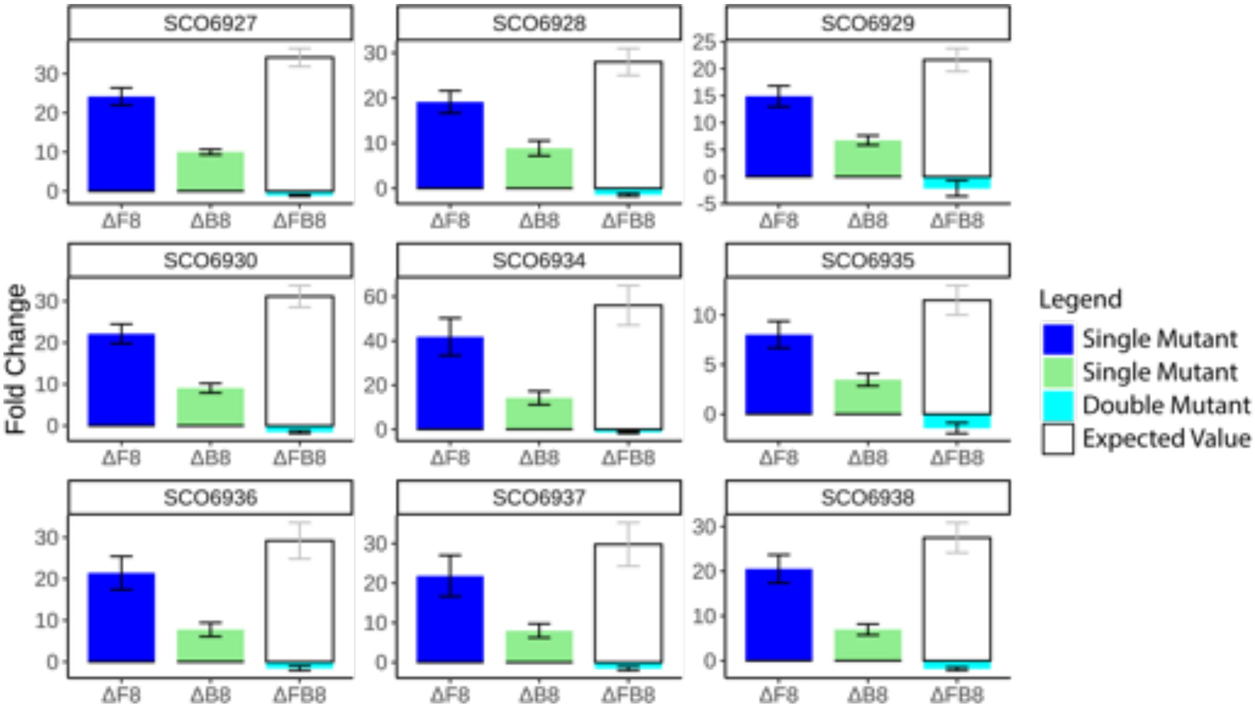

Fig. S14. Epistatic effects on lanthipeptide biosynthetic gene expression by the Actinobacterial G protein systems (AGPSs) components CvnF8 and CvnB8. Fold-change expression data from Nanostring profiling of nine genes within a lanthipeptide biosynthetic gene cluster associated with the CvnF8/CvnB8 AGPS. Bar plots show expression levels in single mutants (*ΔcvnF8*, *ΔcvnB8*) and the *ΔcvnF8ΔcvnB8* double mutant. Hollow bars indicate the expected additive fold-change in the double mutant based on individual mutant effects. Deviations between observed and expected expression in the double mutant reveal epistatic (non-additive) effects of CvnF8 and CvnB8 on regulation of the cluster.

Note: *ΔcvnF8* and *ΔcvnB8* refer to full-gene deletions generated by replacement of the open reading frame with an apramycin resistance cassette.

Table S1. Strains used in this study.

Strain	Relevant Genotype	Use	Reference
E. coli ET12567 / pUZ8002	*dam tra* genes	*Streptomyces* conjugation	MacNeil et al. (1)
E. coli / St1G8	S. coelicolor gDNA: 7674874−7712565 bp	gDNA cosmid with cvn8	Redenbach et al. (2)
E. coli BW25113 / pIJ790	λ RED genes	Recombining the targeting cassette	Gust et al. (3)
E. coli DH5α / pIJ773	*aac(3)IV*, *oriT* (RK2), FRT sites	Apramycin cassette amplification	Gust et al. (3)
E. coli XL1-Blue	*endA*1 *gyrA*96(*nal*^{R}) *thi*-1 *recA*1 *relA*1 *lac* *gln*V4' F'[::Tn10 *proAB*$^{+}$ *lacI*^{q} Δ(*lacZ*) M15] *hsdR*17(r_{K}^{-} m_{K}^{+})	Routine gibson assembly	Berkeley QB3
E. coli BL21 (DE3) / pLC21-ScvnA8	*E. coli* str. B F^{-} *ompT gal dcm lon h sdS*$_{B}$($r_{B}^{-}m_{B}^{-}$) λ(DE3 [*lacI lacUV5*-*T7p07 ind1 sam7 nin5*]) [*malB*$^{+}$]$_{K-12}$(λ^{S})	Protein expression of CvnA8	This study
E. coli BL21 (DE3) / pLC21-Strep-cvnF8	*E. coli* str. B F^{-} *ompT gal dcm lon h sdS*$_{B}$($r_{B}^{-}m_{B}^{-}$) λ(DE3 [*lacI lacUV5*-*T7p07 ind1 sam7 nin5*]) [*malB*$^{+}$]$_{K-12}$(λ^{S})	Protein expression of CvnF8	This study
E. coli BL21 (DE3) / pLC21-ScvnA8(H183A)	*E. coli* str. B F^{-} *ompT gal dcm lon h sdS*$_{B}$($r_{B}^{-}m_{B}^{-}$) λ(DE3 [*lacI lacUV5*-*T7p07 ind1 sam7 nin5*]) [*malB*$^{+}$]$_{K-12}$(λ^{S})	Protein expression of CvnA8(H183A)	This study
S. coelicolor M145 strains			
Wild-type	Wild-type, SCP1−SCP2−		Kieser et al. (4)
*ΔcvnA8*mutant	*cvnA8*::*acc(3)IV*	*cvnA8* disrupted with Apramycin cassette	Bonet et al. (5)

*ΔcvnB8*mutant	*cvnB8*::*acc(3)IV*	*cvnB8* disrupted with Apramycin cassette	Bonet et al. (5)
*ΔcvnC8*mutant	*cvnC8*::*acc(3)IV*	*cvnC8* disrupted with Apramycin cassette	Bonet et al. (5)
*ΔcvnD8*mutant	*cvnD8*::*acc(3)IV*	*cvnD8* disrupted with Apramycin cassette	Bonet et al. (5)
*ΔcvnF8*mutant	*cvnF8*::*acc(3)IV*	*cvnF8* disrupted with Apramycin cassette	Bonet et al. (5)
*ΔcvnAB8*mutant	*cvnA8::FRT, cvnB8::aac(3)IV*	Double knockout; *cvnA8* cassette excised using pCP20; *cvnB8* dis rupted with Apramycin cassette	This study
*ΔcvnAF8*mutant	*cvnA8::FRT, cvnF8::aac(3)IV*	Double knockout; *cvnA8* cassette excised using pCP20; *cvnF8* dis rupted with Apramycin cassette	This study
*ΔcvnFB8*mutant	*cvnB8::FRT, cvnF8::aac(3)IV*	Double knockout; *cvnB8* cassette excised using pCP20; *cvnF8* dis rupted with Apramycin cassette	This study

Table S2. Primers used in this study.

Primer Name	Gene or plasmid construct	Sequence
Primers for generating the PCR targeting cassette		
LC1004 (Fwd)	*cvnB8*	AGGGATCGCTCCAGAAGGGGGAAGGGCCAGTGGATGTGCCCGCCAGCCTCGCAGAGCA
LC1005 (Rev)	*cvnB8*	GCACATCCACTGGCCCTTCCCCCTTCTGGAGCGATCCCTTCAGCCAATCGACTGGCGAG
LC1009 (Fwd)	*cvnF8*	ATGTCACAGCACCCCAGCGACCCCTACCGCACGCCGCAGTCAGCCAATCGACTGGCGAG
LC1010 (Rev)	*cvnF8*	ATCGCCTACGGCTCCCGGGTGGGGGTGTGGAACGGCTTAGCGCCAGCCTCGCAGAGCA
Primers for overexpression vectors		
LCRJ21	*pLC21-Strep-cvnA8(72-506)*	ATGTGGAGCCACCCGCAGTTCGAAAAAACACGCACCGTCGCCGGTG
LCRJ04	*pLC21-Strep-cvnA8(72-506)*	TCAGGGGGTGGGGTGTTCT
LC2101	*pLC21-Strep-cvnA8(72-506)*	AGAACACCCCACCCCCTGAGCTAGCATGACTGGTGGACA
LC2102	*pLC21-Strep-cvnA8(72-506)*	TGCGGGTGGCTCCACATCATATGTATATCTCCTTCTTAAAGTTAAACA
LC2110	*pLC21-Strep-cvnF8*	TGGAGCCACCCGCAGTTCGAAAAAGGTAGCTCACAGCACCCCAGCGAC

BB319	*pLC21-Strep-cvnF8*	TTAGCGGACGGGGGCGCGG
LC2111	*pLC21-Strep-cvnF8*	CCGCGCCCCCGTCCGCTAAGCTAGCATGACTGGTGGACA
KiDo32	*pLC21-ScvnA8(H183A)*	CATGCGCGAGGCCGCCTTGGTCAGCAAG
KiDo33	*pLC21-ScvnA8(H183A)*	GCTGACCAAGGCGGCCTCGCGCATGGCC

References

1. MacNeil DJ, Gewain KM, Ruby CL, Dezeny G, Gibbons PH, MacNeil T. 1992. Analysis of Streptomyces avermitilis genes required for avermectin biosynthesis utilizing a novel integration vector. Gene 111:61–8.

2. Redenbach M, Kieser HM, Denapaite D, Eichner A, Cullum J, Kinashi H, Hopwood DA. 1996. A set of ordered cosmids and a detailed genetic and physical map for the 8 Mb Streptomyces coelicolor A3(2) chromosome. Mol Microbiol 21:77–96.

3. Gust B, Challis GL, Fowler K, Kieser T, Chater KF. PCR-targeted Streptomyces gene replacement identifies a protein domain needed for biosynthesis of the sesquiterpene soil odor geosmin.

4. Kieser T, Bibb MJ, Buttner MJ, Chater KF, Hopwood DA. 2000. Practical streptomyces. Genetics 59.

5. Bonet B, Ra Y, Cantu Morin LM, Soto Bustos J, Livny J, Traxler MF. 2021. The cvn8 Conservon System Is a Global Regulator of Specialized Metabolism in Streptomyces coelicolor during Interspecies Interactions. mSystems 6:e0028121.
