# Supplementary material for: The accessory protein CvnF8 modulates histidine kinase activity in an actinobacterial G protein system in Streptomyces coelicolor
Source: mBio. 2026 May 27;17(7):e00774-26. doi: 10.1128/mbio.00774-26 (PMC13343842; doi:10.1128/mbio.00774-26)
Supplement: Bioinformatic File Guide — Description of supplemental data files. [file mbio.00774-26-s0010.docx]

**Bioinformatic file guide**

**The accessory protein CvnF8 modulates histidine kinase activity in an Actinobacterial G protein system** **in *Streptomyces coelicolor***

Luis M. Cantu Morin, Kilian Dekoninck, Kyung-Yoon Min, and Matthew F. Traxler*

Department of Plant and Microbial Biology, University of California, Berkeley, California, USA

**Files related to bioinformatics data:**

ribo_partitions_tree.txt <-species tree in Fig 2. The tree can be used in a tree viewer such as the Interactive Tree of Life (<https://itol.embl.de/upload.cgi>)

ribo_partition_species_annotation <- file that can be used to label the leaves of ribo_partitions_tree.txt with species names once it has been generated as a tree at the Interactive Tree of Life (https://itol.embl.de/upload.cgi)

ribo_partition_species_tree_with_bootstraps.pdf <- file that contains an image of the ribo_partitions_tree.txt labeled with species names and bootstrap values

Download_901827_File_Manifest.csv <- manifest of downloaded genomes from IMG

IMG-489-031325-metadata.csv <-metadata of files downloaded from IMG

cvnA_tree_IMG_genome_IMG_gene.pdf <-file that contains an image of the tree made by OG0000103_tree.txt, with leaf labels that include the IMG genome number and the the IMG genome number.

cvnA_tree_species_IMG_geneID.pdf<-file that contains an image of the tree made by OG0000103_tree.txt, with leaf labels that include the species name and the IMG genome number.

OG0000103_tree.txt <- Gene tree of CvnA proteins
